# The collective creativity of Drs. Baro, Pooput, and Callaghan

**DOI:** 10.1128/aac.01943-25

**Published:** 2026-03-16

**Authors:** Paul D. Roepe

**Affiliations:** 1 Department of Chemistry, Georgetown University 8368, Washington, DC, USA; 2 Department of Biochemistry & Cellular & Molecular Biology, Georgetown University 8368, Washington, DC, USA; The Children's Hospital of Philadelphia, Philadelphia, Pennsylvania, USA

**Keywords:** *Plasmodium falciparum*

## LETTER

In an article by Junaid et al. (1), the considerable work that three of my students (Drs. Baro, Pooput, and Callaghan) performed in inventing and perfecting approaches used in this paper that are important for its conclusions was not cited. I write to note this out of respect for the years of work that those students devoted to developing those approaches. In particular, the back-translation of the amino acid sequence to yield a yeast codon-optimized *Plasmodium* gene, expression of those back-translated membrane transporter genes in *S. cerevisiae* yeast, subsequent quantitative analysis of those strains vs. antimalarial drugs by spotting of yeast, and then quantitative growth analysis +/− drug to reveal possible roles of membrane transporter isoforms in antimalarial drug resistance phenomena were first published by Nick Baro, Chaya Pooput, and Paul Callaghan from my laboratory starting in 2011 (2–4). Reference to their collective creativity, hard work, and conclusions should have been included.

I also write to mention that although the paper does a wonderful job in identifying new intraerythrocytic digestive vacuolar (DV) membrane transport proteins putatively involved in antimalarial drug resistance phenomena, at least one known to fall within this category and that is known to reside in the DV (e.g., PfAAT) (5) is not found by the methods used in the study and not mentioned in the paper.
